# Movement to outpatient hysterectomy for benign indications in the United States, 2008–2014

**DOI:** 10.1371/journal.pone.0188812

**Published:** 2017-11-30

**Authors:** Gaby Moawad, Emelline Liu, Chao Song, Alex Z. Fu

**Affiliations:** 1 George Washington University, Washington, DC, United States of America; 2 Intuitive Surgical, Inc, Sunnyvale, California, United States of America; 3 Georgetown University Medical Center, Washington, DC, United States of America; National Academy of Medical Sciences, NEPAL

## Abstract

**Introduction:**

The past decade has witnessed adoption of conservative gynecologic treatments, including minimally invasive surgery (MIS), alongside steady declines in inpatient hysterectomies. It remains unclear what factors have contributed to trends in outpatient benign hysterectomy (BH), as well as whether these trends exacerbate disparities.

**Materials and methods:**

Retrospective cohort of 527,964 women ≥18 years old who underwent BH from 2008 to 2014. BH surgical approaches included: open/abdominal hysterectomy (AH), vaginal hysterectomy (VH), laparoscopic hysterectomy (LH), and robotic-assisted hysterectomy (RH). Quarterly frequencies were calculated by care setting and surgical approach. We used multilevel logistic regression (MLR) using the most recent year of data (2014) to examine the influence of patient-, physician-, and hospital-level preoperative factors and surgical approaches on outpatient migration.

**Results:**

From 2008–2014, surgical approaches for LH and RH increased, which coincided with decreases in VH and AH. Overall, a 44.2% shift was observed from inpatient to outpatient settings (*P*<0.0001). Among all outpatient visits MIS increased, particularly for RH (3.6% to 41.07%). We observed increases in the proportion of non-Hispanic Black and Medicaid patients who obtained MIS in 2014 vs. 2008 (P<0.001). Surgical approach (51.8%) and physician outpatient MIS experience (19.9%) had the greatest influence on predicting outpatient BH. Compared with LH, RH was associated with statistically significantly higher likelihood of outpatient BH overall (OR 1.23; 95% CI, 1.16–1.31), as well as in sub-analyses of more complex cases and hospitals that performed ≥1 RH (P<0.05).

**Conclusion:**

From 2008–2014, rates of LH and RH significantly increased. A significant shift from inpatient to outpatient setting was observed. These findings suggest that RH may facilitate the shift to outpatient BH, particularly for patients with complexities. The adoption of MIS in outpatient settings may improve access to disadvantaged patient groups.

## Introduction

Hysterectomy is the most common procedure for women with benign gynecological conditions. Adoption of conservative gynecologic treatments during the past decade, including minimally invasive procedures, have occurred alongside steady declines in inpatient hysterectomies [1–3]. Minimally invasive surgery (MIS) approaches–including laparoscopic hysterectomy (LH), vaginal hysterectomy (VH), and robotic-assisted hysterectomy (RH)–are becoming increasingly common relative to abdominal hysterectomy (AH) even for complex cases, which is supported by improvements in surgical equipment and emerging developments in specialized fellowship training programs [4–5]. The benefits of MIS for benign hysterectomy (BH) have been clearly documented [1–4]. Patients who have undergone MIS experience fewer medical and surgical complications, better quality of life, and substantially lower medical costs overall [2, 5–11]. Major professional societies in gynecology now recommend MIS as a first line to avoid the morbidity of laparotomy [1, 12]. LH and RH in the outpatient setting are generally considered safe and feasible [5, 13–16]. Given the benefits of minimally invasive hysterectomy and the safety and feasibility profile in an outpatient setting, private payers implemented prior authorization for inpatient surgery starting in 2015 [17, 18]. Beginning in 2016, Centers for Medicare & Medicaid Services (CMS) Medicare Part B Outpatient Prospective Payment System substantially increased reimbursement rates for most BH procedures, providing further incentive for outpatient BH [19].

According to two large database studies, AH was the most commonly performed surgical approach for all BH cases and for inpatient BH by first quarter 2010 [20, 21]. However, these studies were unable to capture the increasingly common outpatient hysterectomy. A recent cross-sectional study of State Ambulatory Surgery and Services Databases from 16 states for year 2011 estimated approximately 100,000–200,000 outpatient hysterectomies per year; approximately 81.5% performed laparoscopically and 16% vaginally [22]. To our knowledge, no previous study has examined trends in care setting over time. This is important because one of the purported benefits of MIS is that it may enable more outpatient surgery.

Our objective in the present study was to examine trends in surgical approaches and care settings among patients in the United States (US) who underwent BH from 2008–2014. We conducted a nationwide analysis of hysterectomy trends stratified by inpatient and outpatient settings using a large hospital administrative database of hospitals in the United States. The influence of patient-, physician-, hospital-level preoperative factors, and surgical approaches on patient pathway to inpatient vs. outpatient BH were also explored.

## Materials and methods

### Data and patients

**Fig 1:**
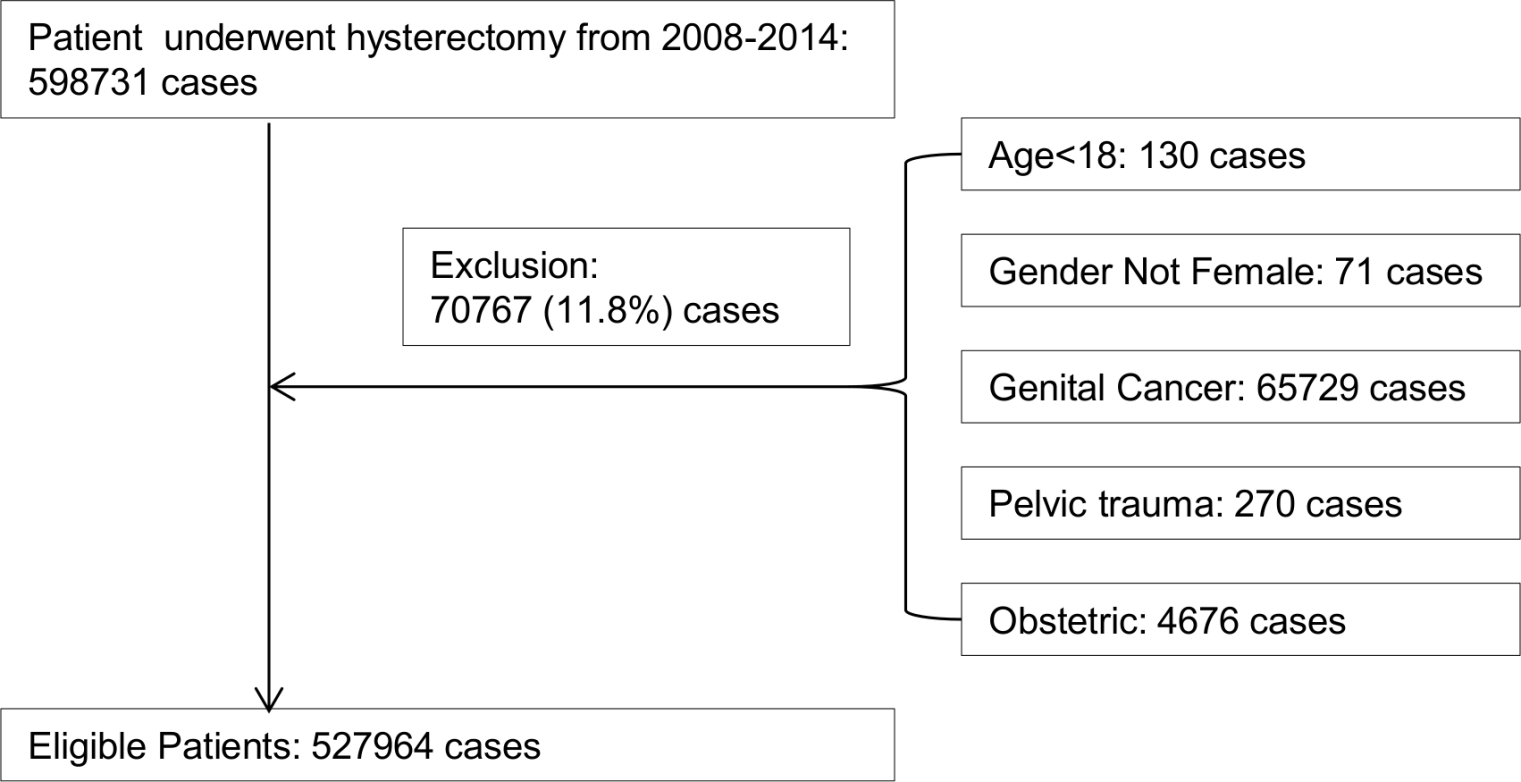
Flow chart of study sample inclusion and exclusion criteria.

The Premier Hospital Perspective^®^ Database (Premier) includes more than 700 hospitals that cover more than 45 million inpatient visits and approximately 210 million outpatient visits from acute care facilities, ambulatory surgery centers, and clinics across the United States [23]. Women aged ≥18 years who underwent BH from 2008 to 2014 were identified in the Premier database. International Classification of Disease, version 9 (ICD-9) procedure codes were used to identify surgical approaches, defined as BH [AH (68.3, 68.39, 68.4, 68.49, 68.9) VH; (68.59); LH (68.31, 68.41, 68.51, V64.41), and RH (17.41, 17.42, 17.44, 17.49, or recorded charged code for robotic instrumentation)]. We excluded 11.8% of women who underwent hysterectomy with: a diagnosis of cancer (ICD-9 CM 179, 180.0, 180.1, 180.8, 180.9, 181, 182.0, 182.1, 182.8, 183.0, 183.2, 183.3, 183.4, 183.5, 183.8, 183.9, 184.0, 184.1, 184.2, 184.3, 184.4, 184.8, 184.9, 233.1, 233.2, 233.3, 233.31, 233.32, 233.39, 236.0, 236.1, 236.2, 236.3); pelvic or lower abdominal trauma (ICD-9 CM 867.4, 867.5, 867.6, 867.7, 867.8, 867.9, 868.00, 868.03, 868.04, 868.09, 868.10, 868.13, 868.14, 868.19, 869.0, 869.1, 879.6, 879.7, 879.8, 879.9, 906.0, 908.1, 908.2, 929.1, 947.4); pregnancy, childbirth, or location in the puerperium (Major diagnosis category 00014)(Fig 1).

### Variables

Inpatient or outpatient BH was defined based on the outpatient/inpatient indicator in Premier (S1 Table). Patients were classified as inpatient if admitted to a hospital, skilled nursing facilities, or long-term care. Patients were classified as outpatients if they spent less than 24 hours in the hospital after surgery, had same day, or ambulatory surgery.

Patient socio-demographic variables included age, race, and insurance type (Medicare, Medicaid, commercial, or self-pay/others); patient clinical characteristics included Charlson Comorbidity Index (CCI), uterine weight (≤250 g vs. >250 g), adhesion (including intra-abdominal and vaginal), obesity (body mass index [BMI] ≥30), indications for surgery (fibroids, endometriosis, uterus bleeding, pelvic prolapse, or chronic pelvic pain) [20, 24], and type of hysterectomy (total or subtotal). Race was included to explore any disparity in the access to healthcare and/or advanced techniques.

The specialty information of physicians prefoming hysterectomy was provided from Premier and was defined as obstetrics and gynecology/OBGYN, gynecologic oncology/GYN ONC, or others. Physician outpatient MIS experience was defined as if the physician had performed at least one outpatient BH through MIS (VH, LH, or RH) since January 1, 2008. Hospital characteristics included location (rural or urban), hospital type (community or teaching), region (Midwest, Northwest, South, or West), and bed size (<200, 200–400, 401–600, or >600). Institutional Review Board (IRB) exemption was obtained from Western Institutional Review Board documenting the analysis of data with no patient, provider, or hospital identifiers.

### Trend analysis

The proportions of the four surgical approaches performed for inpatient vs. outpatient BH were calculated in each quarter. Trend analysis among outpatient BH were performed with further examination of cases with ≥2 complexities (defined as obesity [BMI≥30]), adhesions, or uterine weight >250 g). Since not all hospitals have a robot for RH, the analysis was then limited to hospitals that performed ≥1 RH during 2008–2014, which was used as the proxy for the measure of hospitals with robotic technology for BH. The rates of each surgical approach were calculated by number of quarters after the introduction of RH at a given hospital.

### Statistical analysis

The descriptive analysis of patient, physician, and hospital characteristics observed between 2008 and 2014 were determined by chi-square tests for categorical variables and *t* tests for continuous variables. Trends of each surgical approach within inpatient and outpatient visits were reported. Ordinary least square regression was performed to assess the linear time trend across quarters during the entire timeframe examined. The analyses of inpatients were weighted to obtain nationally representative estimates [25]. No weight was available for the outpatient setting and thus the analyses including outpatient cases were unweighted.

Multivariate logistic regression (MLR) analysis was conducted among women receiving BH to assess the impact of patient, physician, and hospital preoperative factors as well as surgical approach on the odds of the BH being performed in an outpatient vs. inpatient setting [26]. The MLR was conducted using the most recent year of data, 2014. Adjusted R-squared was used for model diagnostics and reported for contributions of variation predicting the likelihood of outpatient BH from different factors. Adjusted odds ratios (ORs) were used to estimate the impact of surgical approach (AH, VH, LH, or RH) on the odds of outpatient BH, using LH as the reference group. Subgroup analyses were conducted among patients with ≥2 complexities and in hospitals having a robot for BH. Additional analysis was repeated using 2008 data to provide a reference for the trend analysis. All analyses were conducted with SAS version 9.4 (SAS Institute, Cary, NC, USA).

## Results

The descriptive characteristics for patients who had a BH in 2008 and 2014 are presented in Table 1. In 2008, AH (44.5%) was the most common surgical approach, and RH was the least common (2.8%). In 2014, LH (31.4%) became the leading surgical approach, followed by RH (29.3%). In both 2008 and 2014, patient characteristics varied significantly across different surgical approaches (all *P* <0.001). Specifically, compared to AH, more patients in the MIS cohorts (ie, patients who underwent VH, LH, and RH) were white and were less likely to have adhesions, obesity, or ≥2 complexities (all *P*<0.0001). The indication of surgery also varied across the four cohorts: AH had more patients with fibroids, and VH had more patients with pelvic prolapse. When comparing changes in patient population between 2008 to 2014, more black, Medicare or Medicare insured, and complex patients had MIS BH in 2014, and substantially more RH procedures were done in nonteaching and in smaller bed size hospitals in 2014. Notably, by analyzing different types of hospitals, both procedure volume and proportion of VH declined substantially among nonteaching hospitals, regardless of bed size.

**Table 1:** Patient characteristics for benign hysterectomy performed in 2008 and 2014.

	Surgical Approach									
	2008 (n = 72,922)					2014 (n = 59,628)				
	AH	VH	LH	RH	*P* value	AH	VH	LH	RH	*P* value
n (%)	32,479 (44.5)	15,327 (21.2)	23,059 (31.6)	2057 (2.8)		15,080 (25.3)	8367 (14.0)	18,700 (31.4)	17,841 (29.3)	
Age, n (%), y					<0.0001					<0.0001
<40	7760 (23.9)	3759 (24.5)	6986 (30.3)	451 (21.9)		2974 (19.7)	1759 (21.0)	5203 (27.8)	4308 (24.6)	
40–44	7905 (24.3)	2857 (18.6)	5652 (24.5)	459 (22.2)		3498 (23.2)	1366 (16.3)	4651 (24.9)	3892 (22.3)	
45–49	8551 (26.4)	2745 (17.9)	5489 (23.8)	546 (26.5)		4050 (26.9)	1272 (15.2)	4124 (22.1)	3797 (21.7)	
50–54	4160 (12.8)	1615 (10.5)	2632 (11.4)	271 (13.2)		2242 (14.9)	837 (10.0)	2220 (11.9)	2276 (13.0)	
55–60	1570 (4.8)	1067 (6.96)	982 (4.3)	129 (6.3)		876 (5.8)	716 (8.6)	965 (5.2)	1157 (6.6)	
≥60	2533 (7.8)	3284 (21.4)	1318 (5.7)	204 (9.9)		1440 (9.6)	2417 (28.9)	1537 (8.2)	2051 (11.7)	
Race, n (%)					<0.0001					<0.0001
White	18,442 (56.8)	10,955 (71.4)	16,089 (69.8)	1476 (71.8)		7765 (51.5)	6205 (74.1)	13,455 (72.0)	12,279 (70.2)	
Black	6670 (20.5)	955 (6.2)	2493 (10.8)	207 (10.1)		4157 (27.6)	670 (8.0)	2656 (14.2)	2578 (14.8)	
Other	2270 (7.0)	907 (5.9)	848 (3.7)	143 (7.0)		4144 (20.9)	1488 (17.8)	2584 (13.8)	2614 (14.5)	
Unknown	5090 (15.7)	2515 (16.4)	3629 (15.7)	231 (11.2)		14 (0.1)	4 (0.1)	11 (0.1)	10 (0.01)	
Insurance type, n (%)					<0.0001					<0.0001
Medicaid	2962 (9.1)	1212 (7.9)	1830 (7.9)	138 (6.7)		2548 (16.9)	1231 (14.7)	2599 (13.9)	1978 (11.3)	
Medicare	2532 (7.8)	2432 (15.9)	1260 (5.5)	181 (8.9)		1485 (9.9)	1800 (21.6)	1497 (8.0)	1776 (10.2)	
Self-pay/other	3186 (9.8)	1424 (9.3)	1883 (8.7)	132 (6.4)		1253 (8.3)	556 (6.7)	1564 (8.4)	1133 (6.5)	
Commercial	23,799 (73.3)	10,259 (66.9)	18,086 (78.4)	1606 (78.7)		9794 (65.0)	4766 (57.0)	13,043 (69.8)	12,594 (72.0)	
CCI, mean (SD)	0.31 (1.0)	0.19 (0.5)	0.19 (0.8)	0.28 (0.8)	<0.0001	0.52 (1.2)	0.26 (0.6)	0.25 (0.7)	0.29 (0.7)	<0.0001
Indication for surgery										
Fibroids (Leiomyma & benign neoplasm)	22,133 (68.2)	5249(34.3)	12,282 (53.3)	1161 (56.4)	<0.0001	11,001 (73.0)	2959(35.4)	10,387 (55.6)	10,100 (57.8)	<0.0001
Endometriosis	8751 (26.9)	2728 (17.8)	7534 (32.7)	591 (28.7)	<0.0001	2792 (18.5)	1880 (22.5)	6622 (35.4)	6221 (35.6)	<0.0001
Pelvic prolapse	2229 (6.9)	8828 (57.6)	3371 (14.6)	175 (7.0)	<0.0001	803 (5.3)	4916 (58.8)	2272 (12.2)	2194 (12.6)	<0.0001
Uterus bleeding	16,240 (50.0)	6655 (43.4)	13,329 (57.8)	1023 (49.7)	<0.0001	7543 (50.0)	3255 (38.9)	10,480 (56.0)	8953 (51.2)	<0.0001
Chronic pelvic pain	5937 (18.3)	1389 (9.1)	5109 (22.2)	376 (18.3)	<0.0001	2709 (18.0)	922 (11.0)	4697 (25.1)	4216 (24.1)	<0.0001
Complexities										
Uterine size >250 g, n (%)	827 (2.6)	470 (3.1)	1477 (6.4)	174 (8.5)	<0.0001	739 (4.9)	567 (6.8)	2469 (13.2)	2676 (15.3)	<0.0001
Adhesions, n (%)	7181 (22.1)	205 (1.3)	339 (14.7)	334 (16.2)	<0.0001	2869 (19.0)	58 (0.7)	1947 (10.4)	2714 (12.1)	<0.0001
Obesity^a^, n (%)	3924 (12.1)	917 (6.0)	1892 (8.2)	212 (10.3)	<0.0001	2787 (18.5)	859 (10.3)	2485 (13.3)	2736 (15.7)	<0.0001
≥2 complexities, n (%)	1320 (4.1)	61 (0.4)	625 (2.7)	77 (3.7)	<0.0001	837 (5.6)	86 (1.0)	854 (4.6)	1031 (5.9)	<0.0001
Type of BH, n (%)					<0.0001					<0.0001
Subtotal	4030 (12.4)	15,324 (100.0)	19,238 (84.2)	1146 (57.0)		2618 (17.4)	8366 (100.0)	10,782 (58.12)	5119 (29.65)	
Total	28,449 (87.6)	3 (0.02)	3610 (15.8)	1146 (43.0)		12,462 (82.6)	1 (0.01)	7768 (41.88)	12,145 (70.4)	
Physician specialty					<0.0001					<0.0001
OBGYN/GYN	29,529 (90.9)	14,603 (92.2)	21,586 (94.5)	1707 (83.9)		12,848 (85.2)	7550 (90.2)	16,476 (88.8)	14,974 (85.7)	
Gynecological oncology	868 (2.7)	544 (3.6)	595 (2.6)	14 (0.7)		881 (5.9)	741 (8.9)	1284 (6.9)	1141 (6.5)	
Other	2082 (6.4)	180 (1.2)	667 (2.9)	313 (15.4)		1223 (8.2)	76 (0.9)	790 (4.3)	1354 (7.8)	
Physicians with outpatient MIS experience	2233 (6.9)	6490 (42.3)	10,674 (46.3)	1008 (51.8)	<0.0001	5927 (39.3)	7235 (86.5)	15,916 (85.1)	16,447 (94.1)	<0.0001
Location					<0.0001					<0.0001
Rural	3485 (10.7)	2275 (14.9)	2596 (11.3)	298 (14.8)		2123 (14.1)	1829 (21.9)	2791 (14.50)	1701 (9.7)	
Urban	28,994 (89.3)	13,052 (85.1)	20,463 (88.7)	1759 (85.2)		12,935 (85.9)	6518 (78.9)	15,830 (85.0)	15,773 (90.3)	
Region					<0.0001					<0.0001
Midwest	5891 (18.1)	3467 (22.6)	4479 (19.3)	594 (28.9)		2832 (18.8)	1481 (17.7)	2936 (15.8)	3545 (20.3)	
Northeast	4411 (13.6)	1456 (9.5)	2381 (10.3)	260 (12.6)		2725 (18.1)	1071 (12.8)	2173 (11.7)	1971 (11.3)	
South	16,887 (52.0)	6391 (41.8)	11,439(49.4)	884 (43.0)		7774 (51.6)	4257 (51.0)	11,047 (59.3)	9908 (56.7)	
West	5290 (16.3)	4013 (26.2)	4760 (20.6)	319 (15.5)		1727 (11.5)	1538 (18.4)	2465 (13.2)	2050 (11.7)	
Teaching hospital					<0.0001					<0.0001
No	21,493 (66.2)	10,813 (70.6)	15,427 (66.9)	1202 (58.4)		8861 (58.9)	5077 (60.8)	12,298 (66.0)	11,375 (65.1)	
Yes	10,986 (33.8)	4514 (29.5)	7632 (33.1)	855 (41.6)		6197 (41.5)	3270(39.2)	6323 (34.0)	6099 (34.9)	
Bed count					<0.0001					<0.0001
<200	4833 (14.9)	2752 (18.0)	4672 (20.1)	186 (9.0)		2714 (18.0)	1794 (21.4)	5025 (26.9)	2591 (14.8)	
201–400	13,242 (40.8)	6036 (39.4)	8183 (35.5)	561 (27.3)		6011 (39.9)	3321 (39.7)	6617 (35.4)	7490 (42.9)	
401–600	8678 (26.8)	4330 (28.3)	6218 (27.0)	845 (41.1)		3988 (26.4)	2145 (25.6)	4125 (22.1)	4653 (26.6)	
>600	5705 (17.6)	2209 (14.4)	4031 (17.5)	465 (22.6)		2367 (15.7)	1107 (13.2)	2933 (15.7)	2747 (15.7)	

^a^BMI ≥30. AH, open/abdominal hysterectomy; BH, benign hysterectomy; BMI, body mass index; CCI, Charlson Comorbidity Index; LH, laparoscopic hysterectomy; MIS, minimally invasive surgery; OBGYN, obstetrics and gynecology; RH, robotic hysterectomy; SD, standard deviation; VH, vaginal hysterectomy, y, year.

**Fig 2:**
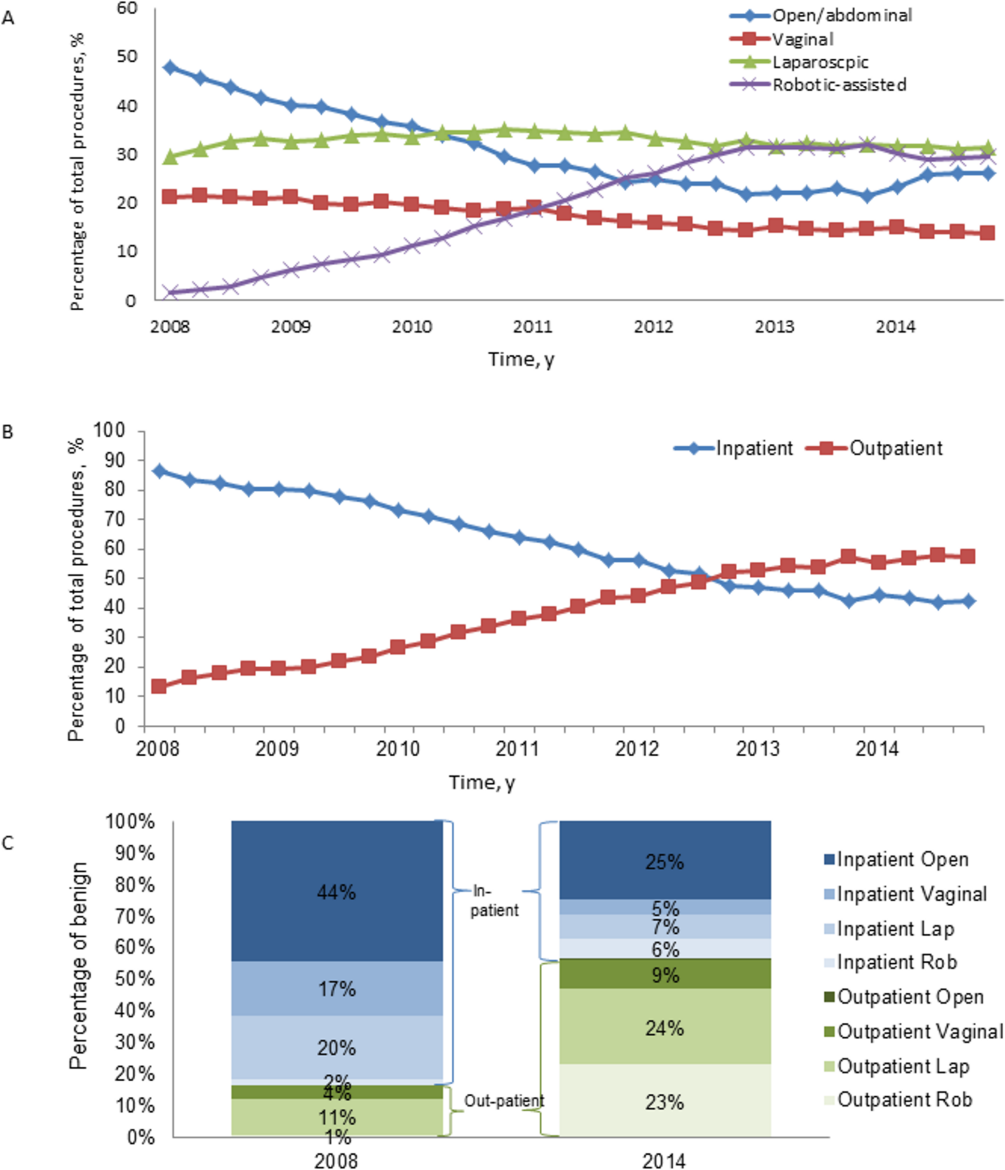
Trends for BH from Q1 2008 through Q4 2014. (A) All Visits by Surgical Approach, (B) All Visits by Care Setting, and (C) All Visits by Surgical Approach and Care Setting, 2008 and 2014 only. BH, benign hysterectomy; RH, robotic hysterectomy; y, year.

From the first quarter (Q1) of 2008 to the last quarter (Q4) of 2014, RH and LH approaches increased (1.7% to 29.3% and 29.4% to 31.2%, respectively), which coincided with decreases in VH and AH (21.2% to 13.5% and 47.8% to 25.9%, respectively; *P*<0.0001; Fig 2A). A statistically significant shift from the inpatient to the outpatient setting was observed; 13.3% of BH were performed as outpatient procedures in 2008 vs. 57.5% in 2014 (*P*<0.0001;Fig 2B; Fig 2C). Among outpatient procedures performed from Q1 2008 to Q4 2014, RH increased from 3.6% to 41.1%, while AH decreased from 2.6% to 0.9%. LH decreased from 69.0% to 42.2%, and VH decreased from 24.8% to 15.8% (S1 Fig; S2 Table). For outpatient surgeries with ≥2 complexities, RH increased from 10.9% to 53.0%, and LH decreased from 78.3% to 42.6% (S2 Fig).

The MLR analysis revealed that in predicting outpatient BH in 2014, patient-level factors contributed 58.99%, surgeon-level factors 20.85%, and hospital-level factors 7.18% (Table 2). Surgical approach (51.84%) and surgeon MIS experience (19.92%) in the outpatient setting were the top two individual contributing factors. Among patient-level factors, subtotal hysterectomy was associated with increased likelihood of outpatient BH; being older, nonwhite, obese, insured by Medicaid, or with a higher CCI was associated with decreased likelihood of outpatient BH (S3 Table). Surgeon with experience in outpatient MIS, OBGYN surgeon, and hospitals in the South region were associated with increased likelihoods of outpatient BH.

**Table 2:** Contributions of hospital, surgeon, and patient characteristics to the variation in predicting likelihood of outpatient BH, 2014.

		All	Obese Population	Adhesion Population	Uterine Weight >250 g Population
Variation %					
Full model		63.11	66.45	63.20	71.59
Patient Characteristics		58.99	61.91	57.21	69.37
	Social-demographic	5.21	5.91	4.75	3.33
	Surgical approach	51.84	52.85	49.61	63.93
	CCI	1.14	1.32	0.89	0.63
	Complexity	9.16	11.01	6.16	3.84
	Obesity	0.74	N/A	0.69	0.88
	Adhesion	3.59	4.31	N/A	3.14
	Uterus weight	5.18	6.76	5.40	N/A
	Indication of surgery	8.44	10.50	6.51	8.22
	Fibroids	0.45	0.38	0.24	4.28
	Endometriosis	6.49	8.88	3.85	3.11
	Pelvic prolapse	0.06	0.00	0.12	0.00
	Uterus bleeding	0.32	0.59	0.17	0.37
	Chronic pelvic pain	1.59	1.54	2.87	0.00
	Total/Subtotal hysterectomy	4.71	3.54	4.42	6.28
Physician characteristics		20.85	22.88	23.4	22.22
	Specialty	1.48	1.81	3.9	0.21
	Physician experience of outpatient MIS	19.92	21.64	19.89	21.79
Hospital characteristics		7.18	8.19	9.63	8.51

BH, benign hysterectomy; CCI, Charlson Comorbidity Index; MIS, minimally invasive surgery.

With regard to the effect of surgical approach (Table 3), compared with the reference group of LH, RH was associated with a modestly increased odds of outpatient BH (OR 1.23; 95% CI, 1.16–1.31). In those patients with complexities, RH was associated with an increased odds of outpatient BH, particularly among patients with adhesions (OR 1.74; 95% CI, 1.49–2.04). Compared to AH, strongly increased odds of outpatient MIS were observed for VH (OR 80.52; 95% CI, 70.29–92.24), LH (OR 111.38; 95% CI, 98.91–125.56), and RH (OR 137.27; 95% CI, 121.69–154.85).

**Table 3:** Estimated effects of surgical approaches on the likelihood of outpatient BH, 2014 (N = 59,628).

OR (95% CI)		All Population*	Obese^†^ (BMI ≥30)	Adhesions^‡^	Uterine Weight >250 g^§^
All hospitals	VH vs. AH	80.52 (70.29–92.24)	89.29 (60.79–131.14)	31.82 (15.41–65.70)	303.16 (160.58–572.33)
	LH vs. AH	111.38 (98.91–125.56)	108.56 (78.13–150.84)	69.31 (48.06–99.95)	575.56 (339.16–976.37)
	RH vs. AH	137.27 (121.69–154.85)	136.99 (98.58–190.38)	120.75 (83.62–174.37)	606.34 (358.23->999.99)
	RH vs. LH	1.23 (1.16–1.31)	1.26 (1.09–1.47)	1.74 (1.49–2.04)	1.05 (0.79–1.40)
Robotic BH hospital	VH vs. AH	74.16 (63.49–86.62)	105.63 (65.91–169.29)	20.94 (8.16–53.70)	233.31 (110.75–491.52)
	LH vs. AH	104.58 (91.11–120.05)	118.51 (78.20–179.59)	61.81 (40.87–93.47)	423.14 (229.96–778.61)
	RH vs. AH	129.27 (112.77–148.19)	160.28 (106.25–241.79)	104.57 (69.40–157.56)	632.74 (346.17->999.99)
	RH vs. LH	1.24 (1.16–1.32)	1.35 (1.15–1.60)	1.69 (1.42–2.02)	1.50 (1.08–2.08)

AH, open/abdominal hysterectomy; BH, benign hysterectomy; BMI, body mass index; CI, confidence interval; g, grams; LH, laparoscopic hysterectomy; OR, odds ratio; RH, robotic hysterectomy; Robotic BH hospital, hospitals with a robot for BH in 2014; VH, vaginal hysterectomy.

*Adjusted for age, race, insurance type, hysterectomy type (total/subtotal), Charlson comorbidity score, indication for surgery (fibroids, endometriosis, pelvic prolapse, uterus bleeding, and chronic pelvic pain), obese, uterine weight (>250 g vs. ≤250 g), physician specialty, teaching, hospital region, hospital area (urban/rural), bed size.

^†^Adjusted for age, race, insurance type, hysterectomy type (total/subtotal), Charlson comorbidity score, indication for surgery (fibroids, endometriosis, pelvic prolapse, uterus bleeding, and chronic pelvic pain), adhesion, uterine weight (>250 g vs. ≤250 g), physician speciality, physician experience of outpatient minimally invasive surgery, teaching, hospital region, hospital area (urban/rural), bed size.

^‡^Adjusted for age, race, insurance type, hysterectomy type (total/subtotal), Charlson comorbidity score, indcation for surgery (fibroids, endometriosis, pelvic prolapse, uterus bleeding, and chronic pelvic pain), obese, uterine weight (>250 g vs. ≤250 g), physician speciality, physician experience of outpatient minimally invasive surgery, teaching, hospital region, hospital area (urban/rural), bed size.

^§^Adjusted for age, race, insurance type, hysterectomy type (total/subtotal), Charlson comorbidity score, indication for surgery (fibroids, endometriosis, pelvic prolapse, uterus bleeding, and chronic pelvic pain), obese, adhesion, physician speciality, physician experience of outpatient minimally invasive surgery, teaching, hospital region, hospital area (urban/rural), bed size.

Stratifying the data for robotic BH hospitals, similar patterns were found (Table 3). In this subgroup of hospitals, the odds of outpatient BH for RH vs. LH was similar to that observed for all hospitals. The analysis of 2008 data shows similar results (S4 Table).

## Discussion

The present study provides the first nationwide analysis of trends for BH stratified by setting and surgical approach. The analyses of the changing trends of surgical approaches in benign hysterectomy–overall, in inpatient and outpatient settings, and among those complex patients–were descriptive. The results demonstrate that from 2008–2014 care setting for BH significantly shifted from inpatient to outpatient, paralleled by the substantial increase of MIS. We further performed a multivariate logistic regression to analyze the contribution or likelihood of in vs. outpatients, by which we confirmed that after controlling other factors, surgical approach of MIS, in particular robotic, increases the likelihood of outpatient surgery. Surgical approach and physician outpatient MIS experience were the top two factors that predicted a patient having an outpatient BH. Investigating the various impacts within different surgical approaches, RH is associated with an increased odds of outpatient BH in all hospitals and in hospitals with RH technology, and the odds are greater in patients with complex BH procedures. This suggests that outpatient migration may reduce disparities, improving access to disadvantaged patient groups and in non-teaching hospitals. Moreover, our analyses of the odds of outpatient surgery suggest that MIS adoption and outpatient migration may be associated.

The landscape of BH has changed dramatically in the past decade with more outpatient BH and more MIS procedures being performed. Our findings suggest that BH significantly shifted from inpatient to outpatient, with 13.3% of all hysterectomies performed as an outpatient procedure in 2008 vs. 57.5% in 2014; and the shift to outpatient BH was paralleled with a substantial increase of MIS, including LH and RH. When looking solely at the inpatient setting, AH was still the most common approach, which is consistent with nationwide-based studies based exclusively on inpatient settings [20, 21]. However, when taking into account both the inpatient and outpatient settings, AH was no longer the predominant procedure, dropping from 49.5% in Q1 2008 to 28.1% in Q4 2014. These findings were consistent with previous reports comparing hysterectomy trends in earlier time periods [21, 27–29].

The uptake of MIS in outpatient BH likely stems from a variety of factors, including: improved patient outcomes through less invasive approaches; MIS training and awareness in gynecologic societies; and more advanced technology, which could allow for the shift of more complex BH to outpatient setting. Our analysis included data from 2008 to 2014, a timeframe in which benefits of MIS have been clearly established, likely resulting from the routine performance of and specialized training in MIS [6, 27, 30, 31]. As a result, the proportions of LH and RH both increased. In particular, since approved for gynecology in 2005 [32], RH has become significantly more widespread in both inpatient and outpatient settings and across different hospital types. Although only 1.7% of all hysterectomies performed in Q1 2008 were RH, RH accounted for 29.3% in Q4 2014. LH also increased from 29.4% to 31.2%. When looking only at outpatient cases, the volumes of LH and RH both increased; however, the proportion of LH decreased, which was paralleled with an increase in the proportion of RH over the study period. Similarly, among outpatient BH cases with ≥2 complexities, the proportion of RH increased while the proportion of LH decreased. This indicates that RH may facilitate the outpatient migration through becoming a valuable resource for complex surgical candidates, allowing them access to the benefits of MIS, for which inpatient AH would be the only choice otherwise. Given that RH has a similar safety profile as LH and VH and better outcomes than AH [1, 3, 4, 8, 11], this shift toward RH may decrease overall morbidity and improve the safety profile of hysterectomy by minimizing the use of AH, enabling more MIS in general, and enabling complex MIS gynecologic procedures in the outpatient setting.

The findings from the trend analyses were supported by the MLR results. When considering all preoperative factors, RH was significantly associated with an increased odds of outpatient BH compared to LH. Overall, the odds were greater for patients with complexities and were greatest for such patients in hospitals with robotic technology. Interestingly, across all subgroups, VH was associated with a decreased odds of an outpatient procedure. Additionally, the substantial decrease of VH, particularly in nonteaching hospitals, suggests that there may be real-world challenges for the utilization of VH, despite recommendations from professional societies [33].

There may be a concern that the implementation of a new technology may be accessible only to certain patient groups, which may acerbate health disparities. This study suggests that racial disparities and socioeconomic barriers in access to LH and RH may exist. In 2014, whites were more likely to have MIS, whereas blacks were more likely to have AH. Commercially insured patients were more likely to have LH or RH, while patients with Medicaid or Medicare insurance were more likely to have AH or VH. However, when compared to the 2008 data, a substantially increased volume and proportion of black and Medicaid or Medicare insured patients had access to LH or RH in 2014. In particular for RH, the volume increase in the health disadvantaged groups was greater for RH than for LH. Further, RH use increased substantially in nonteaching and in smaller bed size hospitals. These data suggest that the adoption of RH, or MIS in general, did not acerbate disparity and access to MIS. Rather, it may improve the access of MIS in disadvantaged patient groups and in nonteaching hospitals. This also further explains the trend of outpatient migration of BH from 2008–2014, as RH is available and accessible to more patients in more places.

Multiple studies comparing RH to LH, AH, and VH found comparable clinical outcomes yet cost advantages to nonrobotic approaches [4, 8, 34, 35]. However, since very few AH were conducted in an outpatient setting, previous studies were often restricted to inpatient settings [20, 21], this assured a fair comparison between AH and other surgical approaches but failed to assess the clinical and economic benefits of MIS in an outpatient setting. The benefits of enabling outpatient BH deserve further research and need to be considered in the cost-effectiveness evaluation of surgical approaches for BH. Secondly, patients’ complexity and surgeon experiences can affect outcomes [36–39], yet it is challenging to control for these factors in outcome comparisons. As indicated in this study, patient characteristics vary substantially among different surgical approaches. A study comparing RH to the other approaches found that patients who underwent RH had more complexities (ie, were older, had higher rates of adhesive disease, and large uteri) [2]. Our results further suggest that different surgical approaches serve different patient populations. Hence, additional research is needed to clarify the clinical and surgical complexity of BH patients and to identify the appropriate surgical approach for specific patients under a particular setting.

Potential indications of open surgery versus MIS currently depend largely on the complexity of the pateitn case, the experience and training of the surgeon, as well as the institutional stand of care. To our knowledge, there are no current clear guidelines to triage for these complex cases. Our results help to elucidate physician and institutional factors that differ for these complex cases in the current US clinical environment. Our paper shows the trends of outpatient benign hysterectomy in the current American healthcare system. The findings from our study may help guide international communities by describing US trends– especially that cost containment is a global theme and outpatient migration of a high volume procedure like benign hysterectomy may help save cost from a societal perspective.

This study has several limitations. First, the study used the Premier database, where the outpatient visits were unweighted. Thus, nationwide estimates–rather than population-based estimates–were presented for outpatient trends. However, since it includes both inpatient and outpatient records, the migration trend can be evaluated within the same database. Second, our analyses relied on administrative codes in the database. During early adoption years, some hospitals may have coded RH as LH, failing to utilize the dedicated RH code. Other coding limitations may exist, e.g., the complex cases and indication for surgery were based upon ICD-9 and/or CPT codes rather than physician’s actual justifications. Third, the data were stratified based upon selected complexities due to the lack of a standardized surgical complexity metric. Future studies could investigate a composite complexity score, containing a broader spectrum of weighted complexity indicators, so that the impact of complexity on outpatient migration can be better evaluated. Fourth, our characterization of hospitals with RH technology may be subject to biases due to factors at the facility level or catchment area level. Additional work may inform factors contributing to adoption of MIS techniques, including adoption of RH. Finally, the regression analysis focused on pre-operative factors; it thus did not include all possible variables such as intra-operative factors and concomitant procedures, which may be of interest but are beyond the scope of this study. Finally, including the complications following MIS, AH, and VH would have been an improvement; however Premier only could trace patients within one hospital. The short-term perioperative and long-term complications after surgery could be not fully captured in the Premier. Complications following benign hysterectomy by techniques and care of settings are the subject of further research.

## Conclusions

A shift in setting for BH from inpatient to outpatient was observed from 2008–2014. In outpatient settings, statistically significant increases in MIS, especially the increase of RH in overall and complex cases, were observed. Utilization of RH as the surgical approach was associated with an increased odds of outpatient BH, particularly for patients with adhesions. Further comprehensive cost-effectiveness analysis of BH surgical approaches incorporating patient pathway of inpatient and outpatient setting and real-world comparative effectiveness of surgical outcomes is warranted. Further, the adoption of MIS in outpatient settings may improve access to disadvantaged patient groups.

## Supporting information

S1 Table Inpatient and outpatient definitions from premier database. SNF, skilled nursing facility; TCU, transitional care unit.
(DOCX)

S2 Table Volume of outpatient benign hysterectomy by surgical approach from Q1 2008 through Q4 2014. AH, open/abdominal hysterectomy; BH, benign hysterectomy; LH, laparoscopic hysterectomy; RH, robotic hysterectomy; VH, vaginal hysterectomy. *% of outpatient BH.
(DOCX)

S3 Table Pre-operative factors and surgical approaches in predicting the likelihood of outpatient benign hysterectomy, 2014. BH, benign hysterectomy; BMI, body mass index; CCI, Charlson Comorbidity Index; CI, confidence interval; g, grams; OBGYN, obstetrics and gynecology; OR, odds ratio.
(DOCX)

S4 Table Estimated effects of surgical approaches on the likelihood of outpatient benign hysterectomy, 2008 (N = 72,622). AH, open/abdominal hysterectomy; BH, benign hysterectomy; BMI, body mass index; CI, confidence interval; g, grams; LH, laparoscopic hysterectomy; OR, odds ratio; RH, robotic hysterectomy; Robotic BH hospital, Hospitals with a robot for BH in 2008; VH, vaginal hysterectomy.
*Adjusted for age, race, insurance type, hysterectomy type (total/subtotal), Charlson comorbidity score, indication for surgery (fibroids, endometriosis, pelvic prolapse, uterus bleeding, and chronic pelvic pain), obese, uterine weight (>250 g vs. ≤250 g), physician specialty, teaching, hospital region, hospital area (urban/rural), bed size.
^†^Adjusted for age, race, insurance type, hysterectomy type (total/subtotal), Charlson comorbidity score, indication for surgery (fibroids, endometriosis, pelvic prolapse, uterus bleeding, and chronic pelvic pain), adhesion, uterine weight (>250 g vs. ≤250 g), physician speciality, physician experience of outpatient minimally invasive surgery, teaching, hospital region, hospital area (urban/rural), bed size.
^‡^Adjusted for age, race, insurance type, hysterectomy type (total/subtotal), Charlson comorbidity score, indication for surgery (fibroids, endometriosis, pelvic prolapse, uterus bleeding, and chronic pelvic pain), obese, uterine weight (>250 g vs. ≤250 g), physician speciality, physician experience of outpatient minimally invasive surgery, teaching, hospital region, hospital area (urban/rural), bed size.
^§^Adjusted for age, race, insurance type, hysterectomy type (total/subtotal), Charlson comorbidity score, indication for surgery (fibroids, endometriosis, pelvic prolapse, uterus bleeding, and chronic pelvic pain), obese, adhesion, physician speciality, physician experience of outpatient minimally invasive surgery, teaching, hospital region, hospital area (urban/rural), bed size. ^¶^In 2008, there were 12384 AH cases observed at hospitals with robotic for BH. 12291 (99.25%) of them were performed in inpatient setting and 93 (0.75%) cases an outpatient setting. There is not enough cases to complete estimation for OR of AH vs. LH.
(DOCX)

S1 Fig Outpatient visits trends for BH by surgical approach from Q1 2008 through Q4 2014. BH, benign hysterectomy; RH, robotic hysterectomy; y, year.
(TIF)

S2 Fig Outpatient visits trends with complexities by surgical approach from Q1 2008 through Q4 2014. BH, benign hysterectomy; RH, robotic hysterectomy; y, year.
(TIF)
