# Supplementary material for: Movement to outpatient hysterectomy for benign indications in the United States, 2008–2014
Source: PLoS One. 2017 Nov 30;12(11):e0188812. doi: 10.1371/journal.pone.0188812 (PMC5708798; doi:10.1371/journal.pone.0188812)
Supplement: S1 Table — SNF, skilled nursing facility; TCU, transitional care unit. (DOCX) [file pone.0188812.s001.docx]

**S1 Table. Inpatient and Outpatient Definitions from Premier Database**

| **Setting of Care** | **Patient Type Description** | **Details** |
| --- | --- | --- |
| Inpatient | Inpatient | Patients admitted to the hospital, excluding SNF, hospice, psychiatric, rehabilitation, and chemical dependency. |
| Inpatient | Skilled Nursing | Patients admitted to SNF and TCU units (considered as inpatients). |
| Inpatient | Long-Term Care | Patients admitted to a long-term care unit for custodial care. |
| Inpatient and Outpatient | Rehabilitation | Patients receiving inpatient rehabilitation and outpatient rehabilitation services such as physical therapy, occupational therapy, cardiac, speech therapy, or respiratory rehabilitation. Patients treated for chemical dependency will be excluded from this category. The encounter can be recurring or single visit. |
| Inpatient and Outpatient | Psychiatric | Patients receiving inpatient and outpatient psychiatric services, recurring or single visit. Partial Hospitalization Program (PHP) are outpatients. |
| Inpatient and Outpatient | Hospice | Patients who receive care through an inpatient hospice unit or outpatient hospice care. |
| Inpatient and Outpatient | Chemical Dependency | Patients receiving treatment for chemical dependency and/or addiction treatment. |
| Outpatient | Same Day Surgery | All outpatient, same day, and ambulatory surgery in which the patient is discharged a few hours after the procedure. Patients that receive a blood or chemotherapy on a one visit basis. |
| Outpatient | Emergency | Patients who receive treatment in and are discharged from the emergency department. |
| Outpatient | Observation | Outpatient observation patients. The patient’s hospital visit was 23 hours or less. |
| Outpatient | Diagnostic Testing | Patients undergoing diagnostic tests not considered presurgical testing, including reference lab, X-ray, etc. |
| Outpatient | Recurring/Series | Patients having recurring visits for services including dialysis, chemotherapy infusion and radiation, excluding psychiatric, chemical dependency, and rehabilitation. |
| Outpatient | Presurgical Testing | Patients undergoing diagnostic testing (X-ray, lab, etc.) prior to surgery (ambulatory or inpatient). This would be used when the surgery was cancelled after the presurgical tests were conducted. |
| Outpatient | Home Health | Patients receiving care or services at home. |
| Outpatient | Clinic | Patients who receive care at a free-standing or private clinic. |
| Inpatient and Outpatient | Other | Any patient types not specifically defined above. |

SNF, skilled nursing facility; TCU, transitional care unit.
