# Supplementary material for: Movement to outpatient hysterectomy for benign indications in the United States, 2008–2014
Source: PLoS One. 2017 Nov 30;12(11):e0188812. doi: 10.1371/journal.pone.0188812 (PMC5708798; doi:10.1371/journal.pone.0188812)
Supplement: S2 Table — AH, open/abdominal hysterectomy; BH, benign hysterectomy; LH, laparoscopic hysterectomy; RH, robotic hysterectomy; VH, vaginal hysterectomy. *% of outpatient BH. (DOCX) [file pone.0188812.s002.docx]

**S2 Table. Volume of Outpatient Benign Hysterectomy by Surgical Approach from Q1 2008 through Q4 2014.**

|  | **Outpatient BH** | | **AH*** | | **VH*** | | **LH*** | | **RH*** | |
| --- | --- | --- | --- | --- | --- | --- | --- | --- | --- | --- |
|  | **N** | **%** | **n** | **%** | **n** | **%** | **n** | **%** | **n** | **%** |
| 2008 Q1 | 2298 | 13.3 | 60 | 2.6 | 570 | 24.8 | 1585 | 69.0 | 83 | 3.6 |
| Q2 | 3051 | 16.4 | 60 | 2.0 | 728 | 23.9 | 2144 | 70.3 | 119 | 3.9 |
| Q3 | 3129 | 17.6 | 55 | 1.8 | 739 | 23.6 | 2167 | 69.3 | 168 | 5.4 |
| Q4 | 3736 | 19.4 | 67 | 1.8 | 860 | 23.0 | 2483 | 66.5 | 326 | 8.7 |
| 2009 Q1 | 3516 | 19.6 | 63 | 1.8 | 867 | 24.7 | 2220 | 63.1 | 366 | 10.4 |
| Q2 | 3856 | 20.1 | 57 | 1.5 | 827 | 21.5 | 2498 | 64.8 | 474 | 12.3 |
| Q3 | 4171 | 22.0 | 53 | 1.3 | 864 | 20.7 | 2679 | 64.2 | 575 | 13.8 |
| Q4 | 4644 | 23.5 | 56 | 1.2 | 1047 | 22.6 | 2837 | 61.1 | 704 | 15.2 |
| 2010 Q1 | 4863 | 26.8 | 65 | 1.3 | 982 | 20.2 | 2923 | 60.1 | 893 | 18.4 |
| Q2 | 5555 | 28.8 | 67 | 1.2 | 1144 | 20.6 | 3270 | 58.9 | 1074 | 19.3 |
| Q3 | 6158 | 31.6 | 67 | 1.1 | 1194 | 19.4 | 3518 | 57.1 | 1379 | 22.4 |
| Q4 | 7127 | 33.9 | 77 | 1.1 | 1354 | 19.0 | 4019 | 56.4 | 1677 | 23.5 |
| 2011 Q1 | 7068 | 36.2 | 52 | 0.7 | 1346 | 19.0 | 3837 | 54.3 | 1833 | 25.9 |
| Q2 | 7820 | 37.7 | 88 | 1.1 | 1429 | 18.3 | 4107 | 52.5 | 2196 | 28.1 |
| Q3 | 7971 | 40.3 | 80 | 1.0 | 1303 | 16.4 | 4079 | 51.2 | 2509 | 31.5 |
| Q4 | 8988 | 43.6 | 70 | 0.8 | 1390 | 15.5 | 4369 | 48.6 | 3159 | 35.2 |
| 2012 Q1 | 9213 | 43.9 | 57 | 0.6 | 1414 | 15.4 | 4384 | 47.6 | 3358 | 36.5 |
| Q2 | 10174 | 47.2 | 75 | 0.7 | 1501 | 14.8 | 4530 | 44.5 | 4068 | 40.0 |
| Q3 | 9818 | 48.5 | 86 | 0.9 | 1369 | 13.9 | 4289 | 43.7 | 4074 | 41.5 |
| Q4 | 11481 | 52.4 | 91 | 0.8 | 1599 | 13.9 | 4933 | 43.0 | 4858 | 42.3 |
| 2013 Q1 | 10459 | 52.9 | 91 | 0.9 | 1578 | 15.1 | 4308 | 41.2 | 4482 | 42.9 |
| Q2 | 10856 | 54.2 | 104 | 1.0 | 1639 | 15.1 | 4511 | 41.6 | 4602 | 42.4 |
| Q3 | 9837 | 53.9 | 66 | 0.7 | 1528 | 15.5 | 4064 | 41.3 | 4179 | 42.5 |
| Q4 | 10422 | 57.6 | 79 | 0.8 | 1603 | 15.4 | 4255 | 40.8 | 4485 | 43.0 |
| 2014 Q1 | 7501 | 55.3 | 56 | 0.8 | 1280 | 17.1 | 3118 | 41.6 | 3047 | 40.6 |
| Q2 | 9036 | 56.7 | 107 | 1.2 | 1434 | 15.9 | 3840 | 42.5 | 3655 | 40.5 |
| Q3 | 8545 | 57.9 | 116 | 1.4 | 1367 | 16.0 | 3599 | 42.1 | 3463 | 40.5 |
| Q4 | 8833 | 57.5 | 83 | 0.9 | 1399 | 15.8 | 3723 | 42.2 | 3628 | 41.1 |

AH, open/abdominal hysterectomy; BH, benign hysterectomy; LH, laparoscopic hysterectomy; RH, robotic hysterectomy; VH, vaginal hysterectomy.

*% of outpatient BH.
