# Supplementary material for: Movement to outpatient hysterectomy for benign indications in the United States, 2008–2014
Source: PLoS One. 2017 Nov 30;12(11):e0188812. doi: 10.1371/journal.pone.0188812 (PMC5708798; doi:10.1371/journal.pone.0188812)
Supplement: S3 Table — BH, benign hysterectomy; BMI, body mass index; CCI, Charlson Comorbidity Index; CI, confidence interval; g, grams; OBGYN, obstetrics and gynecology; OR, odds ratio. (DOCX) [file pone.0188812.s003.docx]

**S3 Table. Pre-operative Factors and Surgical Approaches in Predicting the Likelihood of Outpatient Benign Hysterectomy, 2014.**

| **Overall** |  | **Overall** | | |  | **Robotic Hospital** | | |
| --- | --- | --- | --- | --- | --- | --- | --- | --- |
| **Characteristics** |  | **OR** | **95% CI** | |  | **OR** | **95% CI** | |
|  |  |  | **Lower** | **Upper** |  |  | **Lower** | **Upper** |
| Age | 30–40 vs. 18–30 | 0.75 | 0.65 | 0.86 |  | 0.73 | 0.62 | 0.86 |
|  | 40–50 vs. 18–30 | 0.56 | 0.48 | 0.64 |  | 0.53 | 0.44 | 0.62 |
|  | 50–65 vs. 18–30 | 0.58 | 0.50 | 0.67 |  | 0.55 | 0.46 | 0.66 |
|  | >65 vs. 18–30 | 0.56 | 0.46 | 0.67 |  | 0.52 | 0.42 | 0.64 |
| Race | Black vs. white | 0.64 | 0.59 | 0.69 |  | 0.64 | 0.59 | 0.70 |
|  | Hispanic vs. white | 0.83 | 0.78 | 0.89 |  | 0.92 | 0.85 | 0.99 |
|  | Other vs. white | 0.27 | 0.11 | 0.69 |  | 0.20 | 0.07 | 0.55 |
| Insurance type | Medicaid vs. commercial | 0.91 | 0.84 | 0.97 |  | 0.93 | 0.86 | 1.02 |
|  | Medicare vs. commercial | 1.02 | 0.92 | 1.13 |  | 1.03 | 0.92 | 1.15 |
|  | Self-pay/other vs. commercial | 1.02 | 0.93 | 1.13 |  | 1.10 | 0.99 | 1.22 |
| CCI | 1–2 vs. 0 | 0.97 | 0.91 | 1.03 |  | 0.96 | 0.89 | 1.02 |
|  | 3–5 vs. 0 | 0.53 | 0.43 | 0.67 |  | 0.49 | 0.39 | 0.63 |
|  | >6 vs. 0 | 0.31 | 0.19 | 0.51 |  | 0.33 | 0.20 | 0.56 |
| Adhesion | Yes vs. no | 0.32 | 0.29 | 0.34 |  | 0.32 | 0.29 | 0.34 |
| Obesity | BMI 25–30 vs. BMI <25 | 0.57 | 0.35 | 0.91 |  | 0.44 | 0.27 | 0.73 |
|  | BMI 30–39 vs. BMI <25 | 0.77 | 0.71 | 0.84 |  | 0.75 | 0.68 | 0.82 |
|  | BMI >40 vs. BMI <25 | 0.63 | 0.57 | 0.70 |  | 0.64 | 0.58 | 0.72 |
| Uterus weight | ≤250 g vs. >250 g | 4.62 | 4.16 | 5.13 |  | 4.91 | 4.35 | 5.53 |
| Indication of surgery |  |  |  |  |  |  |  |  |
| Fibroids | Yes vs. no | 1.42 | 1.35 | 1.50 |  | 1.51 | 1.42 | 1.60 |
| Endometriosis | Yes vs. no | 2.66 | 2.51 | 2.81 |  | 2.80 | 2.63 | 2.99 |
| Pelvic organ prolapse | Yes vs. no | 0.69 | 0.65 | 0.75 |  | 0.72 | 0.66 | 0.78 |
| Abnormal uterus bleeding | Yes vs. no | 0.96 | 0.91 | 1.01 |  | 0.96 | 0.90 | 1.02 |
| Chronic pelvic pain | Yes vs. no | 1.59 | 1.49 | 1.70 |  | 1.69 | 1.57 | 1.82 |
| Type of hysterectomy | Subtotal hysterectomy vs. total hysterectomy | 1.25 | 1.18 | 1.32 |  | 1.28 | 1.20 | 1.37 |
| Surgical modality | Abdominal vs. laparoscopic | 0.009 | 0.008 | 0.010 |  | 0.010 | 0.008 | 0.011 |
|  | Vaginal vs. laparoscopic | 0.72 | 0.67 | 0.78 |  | 0.71 | 0.65 | 0.77 |
|  | Robotic vs. laparoscopic | 1.24 | 1.17 | 1.31 |  | 1.24 | 1.16 | 1.32 |
| Specialty | Gynecology vs. others | 1.35 | 1.21 | 1.50 |  | 1.38 | 1.22 | 1.55 |
|  | OBGYN vs. others | 2.17 | 1.88 | 2.50 |  | 2.61 | 2.22 | 3.06 |
|  | Gynecological oncology vs. others | 0.99 | 0.85 | 1.15 |  | 1.01 | 0.86 | 1.18 |
| Physician experience of outpatient MIS | Yes vs. no | 2.06 | 1.92 | 2.20 |  | 2.11 | 1.95 | 2.28 |
| Teaching hospital | Community vs. teaching | 1.24 | 1.17 | 1.32 |  | 1.48 | 1.38 | 1.59 |
| Provider region | Midwest vs. West | 1.40 | 1.29 | 1.52 |  | 0.71 | 0.64 | 0.80 |
|  | Northeast vs. West | 0.46 | 0.42 | 0.50 |  | 0.78 | 0.71 | 0.85 |
|  | South vs. West | 2.44 | 2.26 | 2.62 |  | 1.18 | 1.08 | 1.29 |
| Location | Nonrural vs. rural | 0.92 | 0.85 | 0.98 |  | 1.39 | 1.17 | 1.66 |
| Bed size | <100 vs. >600 | 0.81 | 0.73 | 0.89 |  | 1.29 | 1.17 | 1.42 |
|  | 100–200 vs. >600 | 0.90 | 0.83 | 0.97 |  | 0.40 | 0.36 | 0.44 |
|  | 200–400 vs. >600 | 1.18 | 1.09 | 1.28 |  | 2.19 | 2.02 | 2.37 |
|  | 400–600 vs. >600 | 0.98 | 0.87 | 1.11 |  | 1.03 | 0.94 | 1.13 |

BH, benign hysterectomy; BMI, body mass index; CCI, Charlson Comorbidity Index; CI, confidence interval; g, grams; OBGYN, obstetrics and gynecology; OR, odds ratio.
