# Supplementary material for: Movement to outpatient hysterectomy for benign indications in the United States, 2008–2014
Source: PLoS One. 2017 Nov 30;12(11):e0188812. doi: 10.1371/journal.pone.0188812 (PMC5708798; doi:10.1371/journal.pone.0188812)
Supplement: S4 Table — AH, open/abdominal hysterectomy; BH, benign hysterectomy; BMI, body mass index; CI, confidence interval; g, grams; LH, laparoscopic hysterectomy; OR, odds ratio; RH, robotic hysterectomy; Robotic BH hospital, Hospitals with a robot for BH in 2008; VH, vaginal hysterectomy. *Adjusted for age, race, insurance type, hysterectomy type (total/subtotal), Charlson comorbidity score, indication for surgery (fibroids, endometriosis, pelvic prolapse, uterus bleeding, and chronic pelvic pain), obese, uterine weight (>250 g vs. ≤250 g), physician specialty, teaching, hospital region, hospital area (urban/rural), bed size. †Adjusted for age, race, insurance type, hysterectomy type (total/subtotal), Charlson comorbidity score, indication for surgery (fibroids, endometriosis, pelvic prolapse, uterus bleeding, and chronic pelvic pain), adhesion, uterine weight (>250 g vs. ≤250 g), physician speciality, physician experience of outpatient minimally invasive surgery, teaching, hospital region, hospital area (urban/rural), bed size. ‡Adjusted for age, race, insurance type, hysterectomy type (total/subtotal), Charlson comorbidity score, indication for surgery (fibroids, endometriosis, pelvic prolapse, uterus bleeding, and chronic pelvic pain), obese, uterine weight (>250 g vs. ≤250 g), physician speciality, physician experience of outpatient minimally invasive surgery, teaching, hospital region, hospital area (urban/rural), bed size. §Adjusted for age, race, insurance type, hysterectomy type (total/subtotal), Charlson comorbidity score, indication for surgery (fibroids, endometriosis, pelvic prolapse, uterus bleeding, and chronic pelvic pain), obese, adhesion, physician speciality, physician experience of outpatient minimally invasive surgery, teaching, hospital region, hospital area (urban/rural), bed size. ¶In 2008, there were 12384 AH cases observed at hospitals with robotic for BH. 12291 (99.25%) of them were performed in inpatient setting and 93 (0.75%) cases an outpatient setting. [file pone.0188812.s004.docx]

**S4 Table. Estimated Effects of Surgical Approaches on the Likelihood of Outpatient Benign Hysterectomy, 2008 (N=72,922).**

| **OR (95% CI)** |  | **All Population*** | **Obese^†^ (BMI >30)** | **Adhesions^‡^** | **Uterine Weight  >250 g^§^** |
| --- | --- | --- | --- | --- | --- |
| All hospitals | VH vs. AH | 16.29 (13.94-19.05) | 11.73 (7.10-19.37) | 6.02 (3.14-11.54) | 44.87 (20.31-99.09) |
|  | LH vs. AH | 31.46 (27.20-36.38) | 22.21 (14.21-34.72) | 21.68 (15.06-31.22) | 88.24 (42.10-185.02) |
|  | RH vs. AH | 30.84 (25.92-36.69) | 23.01 (13.20-40.13) | 23.65 (15.05-37.16) | 123.42 (53.31-285.73) |
|  | RH vs. LH | 0.98 (0.87-1.10) | 1.04 (0.70–1.53) | 1.09 (0.80–1.49) | 1.40 (0.88-2.23) |
| Robotic BH hospital | VH vs. AH | 22.86 (14.30-36.54) | ^¶^ | 2.07 (0.13-32.88) | 232.09 (26.63->999.99) |
|  | LH vs. AH | 50.29 (32.15-78.66) | ^¶^ | 31.50 (9.42-105.34) | 211.06 (31.47->999.99) |
|  | RH vs. AH | 54.79 (34.95-85.89) | ^¶^ | 63.30 (18.71-214.10) | 329.98 (48.22->999.99) |
|  | RH vs. LH | 1.09 (0.94–1.27) | 1.47 (0.83–2.60) | 2.01 (1.29-3.14) | 1.53 (0.81-3.44) |

AH, open/abdominal hysterectomy; BH, benign hysterectomy; BMI, body mass index; CI, confidence interval; g, grams; LH, laparoscopic hysterectomy; OR, odds ratio; RH, robotic hysterectomy; Robotic BH hospital, Hospitals with a robot for BH in 2008; VH, vaginal hysterectomy.

*Adjusted for age, race, insurance type, hysterectomy type (total/subtotal), Charlson comorbidity score, indication for surgery (fibroids, endometriosis, pelvic prolapse, uterus bleeding, and chronic pelvic pain), obese, uterine weight (>250 g vs. ≤250 g), physician specialty, physician volume, teaching, hospital region, hospital area (urban/rural), bed size.

^†^Adjusted for age, race, insurance type, hysterectomy type (total/subtotal), Charlson comorbidity score, indication for surgery (fibroids, endometriosis, pelvic prolapse, uterus bleeding, and chronic pelvic pain), adhesion, uterine weight (>250 g vs. ≤250 g), physician speciality, physician experience of outpatient minimally invasive surgery, teaching, hospital region, hospital area (urban/rural), bed size.

^‡^Adjusted for age, race, insurance type, hysterectomy type (total/subtotal), Charlson comorbidity score, indication for surgery (fibroids, endometriosis, pelvic prolapse, uterus bleeding, and chronic pelvic pain), obese, uterine weight (>250 g vs. ≤250 g), physician speciality, physician experience of outpatient minimally invasive surgery, teaching, hospital region, hospital area (urban/rural), bed size.

^§^Adjusted for age, race, insurance type, hysterectomy type (total/subtotal), Charlson comorbidity score, indication for surgery (fibroids, endometriosis, pelvic prolapse, uterus bleeding, and chronic pelvic pain), obese, adhesion, physician speciality, physician experience of outpatient minimally invasive surgery, teaching, hospital region, hospital area (urban/rural), bed size.

^¶^In 2008, there were 12384 AH cases observed at hospitals with robotic for BH. 12291 (99.25%) of them were performed in inpatient setting and 93 (0.75%) cases an outpatient setting. There is not enough cases to complete estimation for OR of AH vs. LH.
