# Supplementary figures and images for: Movement to outpatient hysterectomy for benign indications in the United States, 2008–2014
Source: PLoS One. 2017 Nov 30;12(11):e0188812. doi: 10.1371/journal.pone.0188812 (PMC5708798; doi:10.1371/journal.pone.0188812)

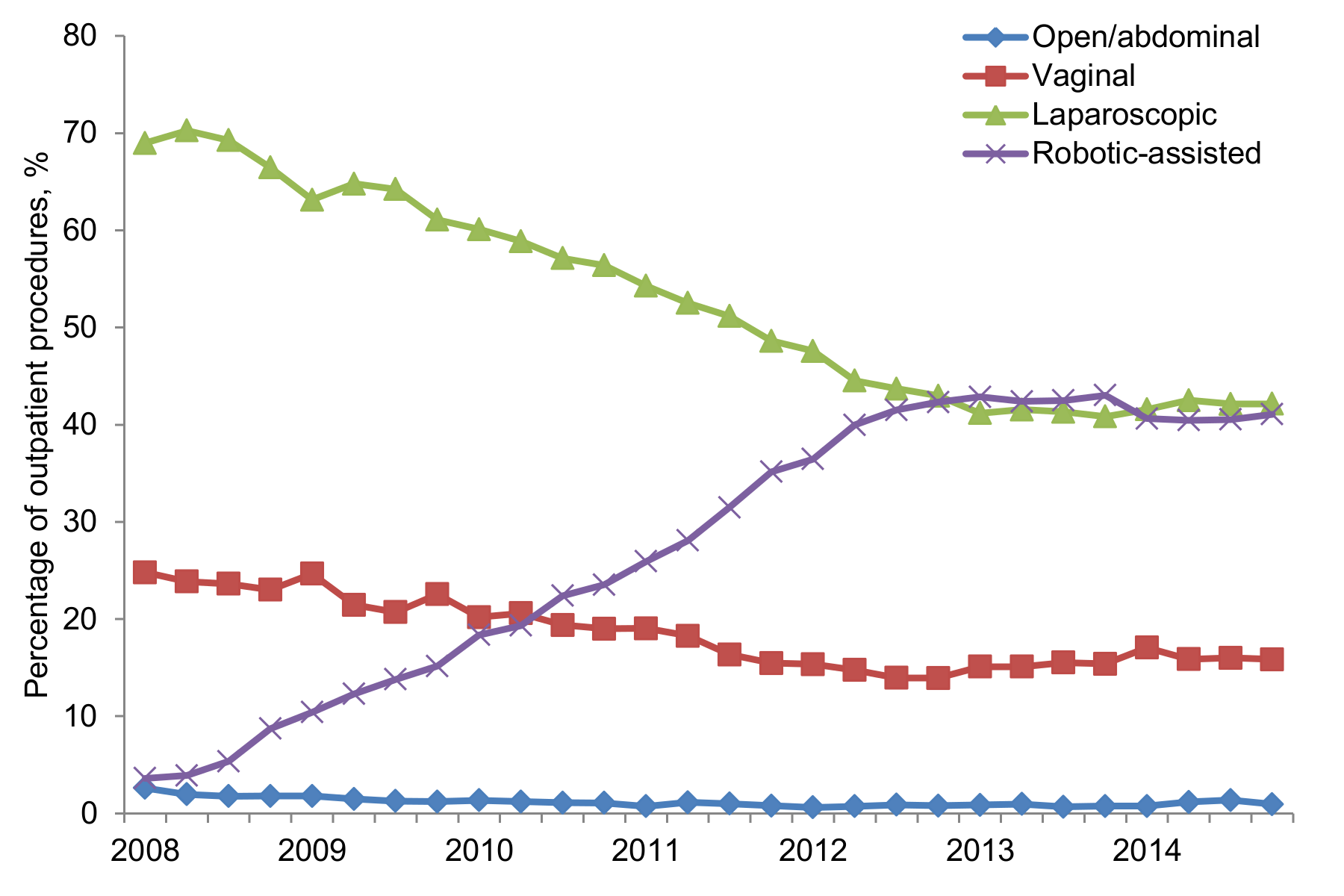

Supplement: S1 Fig — BH, benign hysterectomy; RH, robotic hysterectomy; y, year. (TIF) [file pone.0188812.s005.tif]

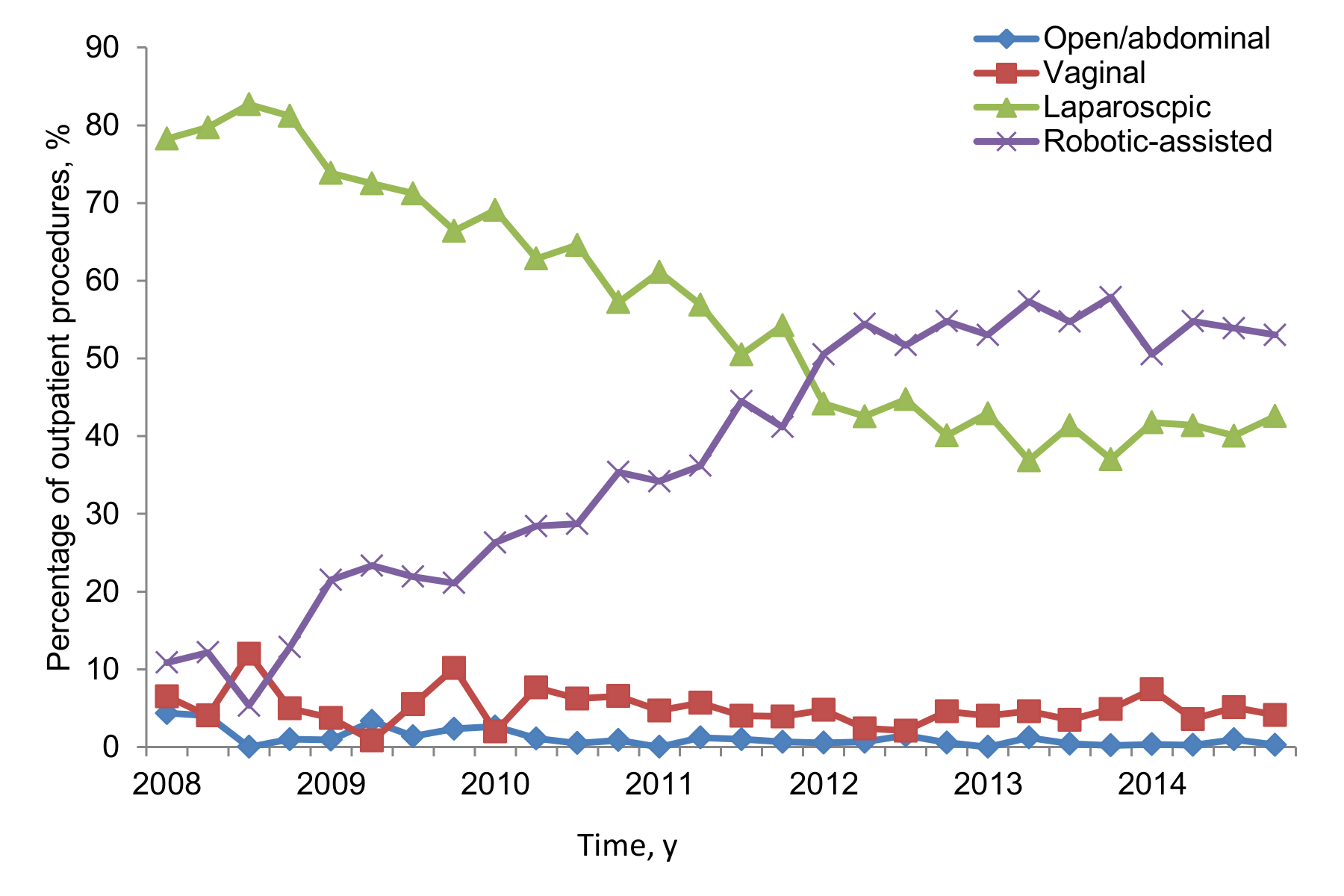

Supplement: S2 Fig — BH, benign hysterectomy; RH, robotic hysterectomy; y, year. (TIF) [file pone.0188812.s006.tif]
